# The Clinical Phenotype of Chinese Patients With Autoimmune Pancreatitis Differs Significantly From Western Patients

**DOI:** 10.3389/fmed.2022.771784

**Published:** 2022-03-07

**Authors:** Qiwen Jin, Yongpeng Ge, Xixia Chen, Chang Tan, Zhenguo Huang, Bei Wang, Bo Zhang, Qinglin Peng, Xiaodi Wang, Guochun Wang

**Affiliations:** ^1^Peking University China-Japan Friendship School of Clinical Medicine, Beijing, China; ^2^Department of Rheumatology, China-Japan Friendship Hospital, Beijing, China; ^3^Department of Radiology, China-Japan Friendship Hospital, Beijing, China; ^4^Department of Pathology, China-Japan Friendship Hospital, Beijing, China; ^5^Department of Ultrasound, China-Japan Friendship Hospital, Beijing, China; ^6^Beijing Key Lab for Immune-Mediated Inflammatory Diseases, Department of Rheumatology, China-Japan Friendship Hospital, Beijing, China; ^7^Department of Gastroenterology, China-Japan Friendship Hospital, Beijing, China

**Keywords:** autoimmune pancreatitis, IgG4, western, Chinese, comparison

## Abstract

**Aim:**

To characterize the clinical features of autoimmune pancreatitis (AIP) in China and compare differences between our Chinese cohort and Western cohorts.

**Methods:**

This was a retrospective study of patients with AIP that was carried out in the China-Japan Friendship Hospital between January 2010 and April 2021. We included a total of 50 patients (46 males and 4 females) aged between 27 and 86 years who fulfilled the international Consensus Diagnostic (ICD) Criteria. For comparative purposes, we included data from seven representative Western cohorts.

**Result:**

When comparing Chinese and Western patients, we found that obstructive jaundice was the most frequent initial symptom (68 vs. 43%, *P* < 0.001). Extra-pancreatic organ involvement was more common in Chinese patients (68 vs. 30%, *P* < 0.001). Sclerosing cholangitis was the most frequent extrapancreatic lesion (48 vs. 24%, *P* = 0.001). The elevation of serum IgG4 was more obvious in our cohort (86 vs. 49%, *P* < 0.001). Conversely, the rates of ANA-positivity were significantly higher in Western populations (17 vs. 50%, *P* = 0.006). With regards to imaging, diffuse swelling was significantly more common in China (44 vs. 27%, *P* = 0.021). Steroid therapy was used more frequently in our Chinese patients (84 vs. 59%, *P* = 0.001). The steroid-response rate was also significantly higher in our Chinese patients (85 vs. 54%, *P* = 0.001); However, the rate of resection was higher in Western cohorts (2 vs. 31%, *P* < 0.001). There was no significant difference between the two populations with regards to recurrence rate (33 vs. 33%, *P* = 1.000).

**Conclusion:**

This study identified significant differences between Chinese and Western populations of patients with AIP. Within the Chinese population, AIP was more likely to have jaundice and extra-pancreatic organ involvement, and elevated serum IgG4 levels. Chinese patients were also showed favorable responses to treatment with glucocorticoids.

## Introduction

In 1995, Yoshida et al. (1) described a case of chronic pancreatitis with impaired pancreatic exocrine function and were the first to refer to this condition as autoimmune pancreatitis (AIP). AIP is considered as a unique type of chronic pancreatitis that is mediated by autoimmunity and characterized by the infiltration of lymphocytes and plasma cells, diffuse enlargement of the pancreas, irregular changes in the pancreatic duct (2, 3), and elevated levels of serum immunoglobulin 4 (IgG4)(4), with or without the involvement of extra-pancreatic organs (5–14). In 2003, pathologists in the United States and Europe reported another unique histological pattern that they referred to as idiopathic duct centric pancreatitis (IDCP) (15); this condition was initially referred to as AIP with granulocyte epithelial lesions (GEL) (16) but is now referred to as type 2 AIP. In 2011, the International Consensus Diagnostic Criteria (ICDC) for AIP was proposed; according to the ICDC, AIP was divided into type 1 AIP (lymphoplasmacytic sclerosing pancreatitis, LPSP) and type 2 AIP (idiopathic duct centric pancreatitis, IDCP) (17).

Thus far, the literature relating to AIP was derived predominantly from Japan and western countries. There have been far fewer systemic studies relating to Chinese patients with AIP. Consequently, very little is known about the characteristics and processes of AIP in Chinese patients. In the present study, we performed a retrospective cohort study of AIP patients and analyzed their demographic, clinical, and laboratory features. Furthermore, we also compared phenotypic differences between our Chinese cohort and patients described in previous western cohorts (18–24).

## Methods

### Patients

We included all AIP patients at the China-Japan Friendship Hospital between January 2006 and April 2021. This study was approved by the China-Japan Friendship Hospital ethics committee board. Written informed consent was obtained from each participant. After their initial visit to the China-Japan Friendship Hospital, we used the medical records system to extract a range of data including clinical manifestations, laboratory data, and imaging studies.

Diagnosis of AIP was based on the 2011 ICDC criteria for AIP (17). Patients with an unknown initial diagnosis, incomplete case data, other infectious diseases, and malignant tumors, were excluded. All patients were followed-up until the end point (April 2021). During follow-up, the response to steroid treatment was defined as an improvement of clinical symptoms and an improvement in imaging and/or laboratory data. Relapse was defined as the reappearance of symptoms with pancreatic and/or extrapancreatic abnormalities on imaging studies (including the bile duct, salivary gland, and retroperitoneum) and/or a marked elevation of serum IgG4 levels (25–28). In our study, the re-elevation of serological levels alone without clinical symptoms, or abnormal imaging, was not considered to represent relapse.

### Histological Examination

Histopathological examinations were performed in 21 AIP patients. Histological examination (hematoxylin and eosin staining) was performed for all tissue samples. We also carried out immunohistochemical staining with anti-CD38, anti-CD138, anti-IgG, and anti-IgG4 antibodies.

### Literature Review

To compare differences in the characteristics of AIP between our Chinese cohort and the western population, we performed literature searches to extract relevant studies from PubMed, EMBASE, and the Cochrane Library. The following search terms were used: “autoimmune pancreatitis,” “AIP,” “immunoglobulin G4,” and “IgG4.” Literature searches were performed between January 2006 and March 2021; articles were extracted manually.

### Statistical Analysis

Data were analyzed using IBM SPSS Statistics version 26.0 (IBM, Armonk, NY, USA). Continuous normally and non-normally distributed quantitative data are presented as mean ± standard deviations (SD) or median (first quartile, third quartile), respectively. Qualitative variables are presented as the number of patients (percentage). Categorical variables were analyzed by Fisher's exact test or the Chi-squared test, as appropriate. A *P* < 0.05 was considered statistically significant.

## Results

### Clinical Characteristics of AIP Patients

A total of 50 Chinese AIP cases were enrolled in this study. The mean age at onset of disease was 61.9 ± 11.3 years and the median duration of symptoms before diagnosis was 1.75 (range: 1.0–6.0) months. Males accounted for 92% of the study population. According to the 2011 ICDC criteria for AIP, there were 44 (88%), 2 (4%), 2 (4%), and 2 (4%) patients diagnosed as definitive type 1 AIP, probable type 1 AIP, probable type 2 AIP and AIP-not otherwise specified, respectively. None of the patients were clearly diagnosed as with definitive type 2 AIP.

Clinical manifestations are shown in Table 1. The most common initial symptoms were jaundice (68%) and weight loss (68%), followed by abdominal pain (52%). Only one patient presented with fatty diarrhea (2%). Nausea and vomiting, and swelling of the submandibular gland, presented in 4 (8%) and 5 (10%) patients. Only 3 (6%) patients presented with a history of allergic disease (allergic rhinitis or bronchial asthma). Thirty-four (68%) patients presented with extra-pancreatic organ involvement. The most common organs involved were the biliary duct (48%), salivary and lacrimal glands (26%), and the lymph nodes (18%). Other manifestations included lung disease (10%), kidney involvement (10%), and retroperitoneal fibrosis (6%).

**Table 1:** Comparison of clinical manifestations in different cohorts.

**Parameter, *n/N* (%)**	**Current cohort**	**Western cohorts**	***P*-value**
Male	46/50 (92)	239/369 (65)	**<0.001**
**Initial symptoms**			
Obstructive jaundice	34/50 (68)	182/422 (43)	**<0.001**
Weight loss	34/50 (68)	72/422 (17)	**<0.001**
Abdominal pain	26/50 (52)	172/422 (41)	0.128
Swelling of the	5/50 (10)	–	
submandibular gland			
Nausea and vomiting	4/50 (8)	–	
Fatty diarrhea	1/50 (2)	9/422(2)	1.000
**Extra-Pancreatic**	34/50 (68)	100/335 (30)	**<0.001**
**organ involvement**			
Biliary duct	24/50 (48)	50/213 (24)	**0.001**
Salivary and lacrimal glands	13/50 (26)	20/213 (9)	**0.001**
Lymph nodes	9/50 (18)	1/213 (1)	**<0.001**
Lung disease	5/50 (10)	1/213 (1)	**<0.001**
Kidney involvement	5/50 (10)	2/213 (1)	**<0.001**
Retroperitoneal fibrosis	3/50 (6)	7/213 (3)	0.623
Thyroid	0/50 (0)	5/213 (2)	0.604
**Serological findings**			
Elevation of serum IgG	28/40 (70)	8/21 (38)	**0.027**
Elevation of serum IgG4	38/44 (86)	124/251 (49)	**<0.001**
Positive ANA	8/46 (17)	10/20 (50)	**0.006**

*–, Not reported; IgG, immunoglobulin G; IgG4, immunoglobulin G4; ANA, antinuclear antibodies*.

To compare with Western AIP patients, we searched the existing literature for cohort studies related to AIP that were reported from the United States and Europe. Finally, we selected seven cohort studies (18–24) with relatively large sample sizes and complete sets of patient information. We merged the data from these seven cohorts and compared this data with our cohort (Tables 1, **3**, **4**). We found that the proportion of AIP patients in our Chinese cohort featured a significantly higher number of males (*P* < 0.001). The frequency of jaundice and weight loss was also significantly higher in our Chinese cohort than in the western cohorts (both *P* < 0.001).

Extra-pancreatic organ involvement was significantly more common in our Chinese cohort. The involvement of the bile duct, salivary and lacrimal glands, lymph nodes, lungs and kidneys were significantly more frequent in our Chinese cohort than in the Western cohorts (*P* ≤ 0.001 for all). However, there was no statistical difference between the two cohorts with respect to retroperitoneal fibrosis or thyroid involvement (P=0.623,0.604, respectively) (Table 1).

Table 2 shows the baseline serological findings from our Chinese cohort of AIP patients. Elevations of Eosinophil (Eos)% and Carbohydrate antigen 199 (CA199)were observed in 14 (29%) and 29 (60%) of patients, respectively. Serum levels of total IgE were elevated in all seven patients examined. Twenty-eight patients (70%) showed an increased serum level of IgG while 38 patients (86%) had an elevated serum level of IgG4. Notably, an increase in serum the serum level of IgG4 > 2× the upper limit of the normal range was observed in 34 (77%) AIP patients. ANAs were positive in eight of the 46 patients (17%), among them, 5 patients were nuclear speckled patterns, 2 patients were cytoplasmic speckled patterns and 1 case was spindle pattern. Antinuclear antibodies (ANCAs) were positive in two of the 39 patients (5%), both of them were PR3-ANCA positive. The median Eos%, IgE level, CA19-9 level, IgG level, and IgG4 level were 4.50%, 326 IU/ml, 45.56 U/ml, 1,850 mg/dl, and 1,050 mg/dl, respectively.

**Table 2:** Baseline serological characteristics of AIP patients.

**Parameter (normal range)**	***n/N* (%)**	**Median (quartile)**
Elevated Eos% (0.4–8%)	14/49 (29)	4.50 (2.15, 8.65)
Elevated IgE level (5–161 IU/ml)	7/7 (100)	326 (211, 1,080)
Elevated CA199 level (<27.00 U/ml)	29/48 (60)	45.56 (14.38, 180.13)
Elevated IgG level (694–1,620 mg/dl)	28/40 (70)	1,850 (1,510, 2,190)
Elevated IgG4 level (3.0–201 mg/dl)	38/44 (86)	1,050 (463, 1,410)
Serum IgG4 level > 2 × Upper limit of range	34/44(77)	
Positive ANA	8/46 (17)	
Positive ANCA	2/39 (5)	

*Eos%, eosinophil%; IgE, immunoglobulin E; CA199, carbohydrate antigen 199; IgG, immunoglobulin G; IgG4, immunoglobulin G4; ANA, antinuclear antibodies; ANCA, anti-neutrophil cytoplasmic antibodies*.

When compared with the western cohort, the elevation of both serum IgG levels and IgG4 levels were significantly more frequent in our Chinese cohort (*P* = 0.027 and <0.001, respectively). The positive rate of ANA was also significantly higher in the Chinese cohort than in the western cohorts (*P* = 0.006; Table 1).

### Imaging Features of AIP

Analysis of the imaging data revealed diffuse swelling in 22 (44%) patients, segmental swelling in 25 (50%) patients, stricture of the main pancreatic duct in 5 (10%) patients. Biliary stricture was present in 42 (84%) patients while involvement of the peripancreatic vessels was only evident in 2 (4%) patients.

Compared with the Western cohorts, we found that diffuse swelling of the pancreas was more frequent in our Chinese cohort (*P* = 0.021). However, stricture of the main pancreatic duct was significantly more frequent in the Western cohorts (41%) than that in our Chinese cohort (10%) (*P* < 0.001). Only two patients showed involvement of the peripancreatic vessels in our Chinese cohort; one involved the splenic vein, the other involved the superior mesenteric vein; these were less common than in the Western cohorts (*P* = 0.01; Table 3).

**Table 3:** Comparison of imaging features in different cohorts.

**Image findings, *n/N* (%)**	**Current cohort**	**Western cohorts**	***P*-value**
Diffuse swelling	22/50 (44)	43/161 (27)	**0.021**
Segmental swelling	25/50 (50)	52/92 (57)	0.456
Stricture of main pancreatic duct^*^	5/50 (10)	29/70 (41)	**<0.001**
Biliary stricture^**^	42/50 (84)	20/26 (77)	0.658
Peripancreatic vessel involvement	2/50 (4)	6/26 (23)	**0.01**

* *Diffuse or segmental irregular stricture of the main pancreatic duct*.

** *Dilatation of the intrahepatic and extrahepatic bile duct, thickening of the distal wall of the common bile duct and stricture of the lumen*.

### Pathological Features of AIP

Twenty-one (42%) patients underwent histological evaluation and some patients received biopsy more than once. Of these, pancreatic histology was available for 14 patients [surgical resection specimen (*n* = 1), pancreatic biopsy using endoscopic ultrasonography-fine needle aspiration (*n* = 12), cell brush for smear examination in ERCP (*n*=1)]. Other pathological evidence obtained included lymph node biopsy or resection (*n* = 5), salivary gland biopsy or resection (*n* = 4), liver puncture (*n* = 1), renal puncture (*n* = 1), lung puncture (*n* = 1), and bone marrow puncture (*n* = 1). The most common findings were lymphocytic and plasma cell infiltration. IgG4 immunostaining showed IgG4^+^ cells in 13 out of 21 patients. Only one surgically resected specimen and two biopsy specimens confirmed lymphoplasmacytic sclerosing pancreatitis (LPSP) and showed massive lymphocyte and plasma cell infiltration. IgG4 immunostaining showed a marked increase in the number of IgG4^+^ plasma cells (>10 plasma cells/) and the ratio of IgG4^+^/IgG^+^ cells >40%.

### Treatment and Outcome

One patient underwent the Whipple procedure for suspected pancreatic adenocarcinoma. Forty-two (84%) patients received glucocorticoids as the initial treatment. The usual regimen was 0.5–0.8 mg/kg/d of oral prednisone for 1 month followed by a taper of 5 mg/wk. Three patients received glucocorticoids combined with immunosuppressive therapy such as iguratimod, cyclophosphamide and azathioprine, 15 patients underwent Endoscopic Retrograde Cholangiopancreatography (ERCP) to insert plastic biliary stents due to obstructive jaundice. The remaining four patients were given supportive treatment due to their age or refusal to use glucocorticoids.

By the end of observation (April 2021), 33 AIP patients had undergone follow-up; the median follow-up was 38 (range 1,121) months. The steroid-response rate reached 85% and 11 patients relapsed with an overall recurrence rate of 33%; only 1 patient died from cardiovascular accidents. Twelve AIP patients withdrew (36%). Among the patients with recurrence, 7 patients were re-treated with glucocorticoids, 3 patients were treated with immunosuppressive agents, such as cyclophosphamide, azathioprine, and tacrolimus on the basis of steroids, and 1 patient was a recent recurrence and had not yet seen the doctor. The 10 relapsed patients showed improvement after treatment; of these, two patients had stopped taking the drugs (6, 24 m), and four patients were receiving prednisone dose <5 mg/d for maintenance therapy.

Compared to the Western cohorts, glucocorticoids therapy was the major therapeutic strategy in our cohort (*P* = 0.001). However, the rate of resection was significantly higher in the Western cohorts (31%) than in our Chinese cohort (2%) (*P* < 0.001). The steroid-response rate was significantly higher than in the Western cohorts (*P* = 0.001); the recurrence rate was not significantly different when compared between the two cohorts (*P* = 1.000; Table 4).

**Table 4:** Comparison of therapy and prognosis between different cohorts.

**Parameter, *n/N* (%)**	**Current cohort**	**Western cohorts**	***P*-value**
**Treatment**			
Glucocorticoids therapy	42/50 (84)	181/308 (59)	**0.001**
Surgical resection	1/50 (2)	94/308 (31)	**<0.001**
**Steroid-Response rate**	28/33 (85)	90/168 (54)	**0.001**
**Recurrence rate**	11/33 (33)	102/307 (33)	1.000
**Steroid-Withdrawal rate**	12/33 (36)	–	

*–, Not reported*.

## Discussion

To our knowledge, this study is the first to compare a range of clinical features between Eastern (our Chinese cohort) and Western patients with AIP. We found that the proportion of males in the Chinese AIP cohort was higher, jaundice, and extra-pancreatic organ involvement was more common, the serum level of IgG4 was significantly increased, and the steroid-response rate was higher.

We found that the mean onset age of AIP was similar to western cohorts with a predominance in elderly people. However, the proportion of males (92%) was significantly higher in our cohort than in the Western population, this feature is also showed in other Chinese studies (29–31). Furthermore, we also found that the proportion of male patients in our center was higher than that in other Asian countries, such as in Japan. There was a latest nationwide survey of AIP in Japan (32), it showed that the male-to-female sex ratio was 2.94. The reasons for the different prevalence of AIP in males between China and other countries are unclear and further investigation is needed.

With regards to initial symptoms, the most common clinical symptom was obstructive jaundice; this was usually caused by pancreatic inflammation affecting or compressing the bile ducts, followed by weight loss and abdominal pain, this is consistent with the Chinese cohort in Meng's et al. (33). This can also be explained by frequency of biliary stricture on imaging in patients with AIP. Meanwhile, most of our Chinese patients also had multi-organ involvement; this feature was very obvious when compared with Western cohorts. The most common extra-pancreatic organ involvement was the bile duct, followed by inflammation of the salivary and lacrimal glands, and lymphadenopathy (Table 1). Elevated IgG4 levels were noted more frequently in the Eastern cohort, this indicating that patient demographics (e.g., ethnic background) and referral bias may inevitably have skewed study populations toward those with higher serum levels.

Many AIP patients in our cohort showed typical diffuse enlargement of the pancreas; the rate of stricture of the main pancreatic duct was significantly lower in the Chinese cohort than in the Western cohort. This might explain the high frequency of surgical resection due to the suspicion of pancreatic cancer in the Western cohort. In addition, our study also observed a small number of patients with peripancreatic vascular invasion, as reported in previous studies (19), another Chinese study (34) reported that 46% patients with AIP showed peripancreatic vascular involvement. Due to the lack of surgical biopsies in such patients, the exact cause of this finding remains unclear. We speculate that pancreatic and peri-pancreatic inflammation could involve the blood vessels, thus causing thickening of the tube walls or the formation of luminal thrombosis.

Histologically, most studies confirmed that AIP patients are mainly type 1 AIP (LPSP), while case of type 2 (IDCP) are relatively rare. However, in our study, only a few patients were pathologically diagnosed with LPSP; this may be related to the limited quantity of tissue obtained for pancreatic specimens taken from the core of the pancreas in our cohort. In this case, clinicians rely on clinical manifestations and increased serum IgG4 levels to improve the diagnostic rate. Nevertheless, histological findings remain the gold standard for the diagnosis of AIP, as documented in other studies (31). The main histological findings were interstitial fibrosis and the infiltration of lymphocytes and plasma cells.

In terms of treatment, 84% received glucocorticoids therapy only; 85% had an effective steroid response. Meng et al. (33) reported 80% AIP patients in China received steroid treatment,96% patients got remission after steroid therapy. In contrast, the proportion of patients receiving surgery in Western countries was significantly higher than that in our Chinese cohort; we believe that this is related to the imaging differences of AIP patients in the Western population, who are more likely to have main pancreatic duct stricture, thus mimicking malignant tumors. As AIP patients generally have good initial treatment effects when treated with steroids, immunosuppressants were seldom used in the initial treatment in our study; however, the combination of immunosuppressants was considered in the case of relapse.

In conclusion, our study is the first to summarize the differences in clinical phenotypes among Chinese and Western AIP populations. The reason caused the differences between the eastern and western AIP phenotype is unclear, which may include the genetic background, environmental factor, dietary factors (35). However, there are some limitations of the study that need to be considered. First, this study was a retrospective study and may have led to recall bias caused by incomplete data. Secondly, we identified seven articles from the literature to represent the Western AIP population. Although quality control was carried out in the selection process, it is still difficult to avoid data deviation caused by the analysis of secondary data. Third, the inclusion criteria applied in this study were based on the 2011 ICD criteria; the use of different criteria in the seven studies identified in the literature may have led to different results. Based on these factors, we hope to conduct more prospective studies in China to determine whether these features are unique and have prognostic significance.

## Data Availability Statement

The original contributions presented in the study are included in the article/supplementary materials, further inquiries can be directed to the corresponding author/s.

## Ethics Statement

The studies involving human participants were reviewed and approved by the research ethics board of China-Japan Friendship Hospital. Written informed consent for participation was not required for this study in accordance with the national legislation and the institutional requirements.

## Author Contributions

QJ and GW contributed to conception and design of the study. QJ organized the database, performed the statistical analysis, and wrote the first draft of the manuscript. All authors contributed to manuscript revision, read, and approved the submitted version.

## Conflict of Interest

The authors declare that the research was conducted in the absence of any commercial or financial relationships that could be construed as a potential conflict of interest.

## Publisher's Note

All claims expressed in this article are solely those of the authors and do not necessarily represent those of their affiliated organizations, or those of the publisher, the editors and the reviewers. Any product that may be evaluated in this article, or claim that may be made by its manufacturer, is not guaranteed or endorsed by the publisher.
